# Supplementary material for: Comparative genomics provides new insights into the diversity, physiology, and sexuality of the only industrially exploited tremellomycete: Phaffia rhodozyma
Source: BMC Genomics. 2016 Nov 9;17:901. doi: 10.1186/s12864-016-3244-7 (PMC5103461; doi:10.1186/s12864-016-3244-7)
Supplement: Additional file 6: — List of orphan genes with links to PFAM (related to Additional file 1: Table S1). (ZIP 1428 kb) [file 12864_2016_3244_MOESM6_ESM.zip › PFAM_UNIQUE/G03005_P.html]

#                                                                            --- full sequence --- -------------- this domain -------------   hmm coord   ali coord   env coord
  
# target name        accession   tlen query name           accession   qlen   E-value  score  bias   #  of  c-Evalue  i-Evalue  score  bias  from    to  from    to  from    to  acc description of target
  
#------------------- ---------- ----- -------------------- ---------- ----- --------- ------ ----- --- --- --------- --------- ------ ----- ----- ----- ----- ----- ----- ----- ---- ---------------------
  
G03005\_P             -            394 AT\_hook              PF02178.14    13       0.6    8.7  28.4   1   3      0.02        15    4.4   3.2     1    12    59    70    59    71 0.89 - AT hook motif  
G03005\_P             -            394 AT\_hook              PF02178.14    13       0.6    8.7  28.4   2   3   4.1e-05     0.031   12.7   3.7     1    11    83    93    83    95 0.89 - AT hook motif  
G03005\_P             -            394 AT\_hook              PF02178.14    13       0.6    8.7  28.4   3   3     0.052        39    3.1   5.8     2    11   108   117   107   119 0.84 - AT hook motif  
G03005\_P             -            394 Ten\_N                PF06484.7    370       7.8    4.2   9.7   1   2      0.68     1e+03   -2.8   0.9   235   264    27    56     9    79 0.55 - Teneurin Intracellular Region  
G03005\_P             -            394 Ten\_N                PF06484.7    370       7.8    4.2   9.7   2   2   8.6e-05      0.13   10.0   2.2   157   198   287   328   263   382 0.80 - Teneurin Intracellular Region
